# Claw lesion status in Brazilian commercial sow herds from 2013 to 2023

**DOI:** 10.3389/fvets.2024.1400630

**Published:** 2024-07-29

**Authors:** Ton Kramer, Alyssa S. Cornelison, Alan Klein, Mike T. Socha, Christof Rapp, Lucas A. Rodrigues, Geraldo C. Alberton

**Affiliations:** ^1^Programa de Pós-graduação em Ciências Veterinárias, Universidade Federal do Paraná—Setor Palotina, Palotina, Brazil; ^2^Zinpro Corporation, Eden Prairie, MN, United States

**Keywords:** claw lesions, cluster, lameness, partial least square regression, sows

## Abstract

**Introduction:**

Claw lesions significantly contribute to lameness, greatly affecting sow welfare. This study investigated different factors that would impact the severity of claw lesions in the sows of Brazilian commercial herds.

**Methods:**

A total of 129 herds (*n* = 12,364 sows) were included in the study. Herds were in the Midwest, Southeast, or South regions of Brazil. Inventory sizes were stratified into 250–810 sows, 811–1,300 sows, 1,301–3,000 sows, and 3,001–10,000 sows. Herds belonged to Cooperative (Coop), Integrator, or Independent structures. The herd management was conducted either maintaining breeds from stock on-site (internal), or through purchase of commercially available genetics (external). Herds adopted either individual crates or group housing during gestation. Within each farm, one randomly selected group of sows was scored by the same evaluator (two independent experts evaluated a total of 129 herds) from 0 (none) to 3 (severe) for heel overgrowth and erosion (HOE), heel-sole crack (HSC), separation along the white line (WL), horizontal (CHW) and vertical (CVW) wall cracks, and overgrown toes (T), or dewclaws (DC) in the hind legs after parturition. The study assessed differences and similarities between herds using Principal Component Analysis (PCA) and Hierarchical Agglomerative Clustering (HAC) analysis. The effects of factors (i.e., production structure, management, housing during gestation, and region) were assessed using the partial least squares method (PLS).

**Results and discussion:**

Heel overgrowth and erosion had the highest prevalence, followed by WL and CHW, while the lowest scores were observed for T, DC, and CVW. Herds were grouped in three clusters (i.e., C1, C2, and C3). Heel overgrowth and erosion, HSC, WL, CHW, CVW, and T were decreased by 17, 25, 11, 25, 21, and 17%, respectively, in C3 compared to C1 and 2 combined. Independent structure increased the L-Index in all three clusters. Furthermore, individual housing increased the L-Index regardless of the cluster. The results suggest that shifting toward larger, more technologically advanced herds could potentially benefit claw health. Additionally, adopting group gestation housing appears to mitigate the adverse effects on claw health, although further validation is necessary, as Brazil has only recently transitioned from individual housing practices.

## 1 Introduction

Lameness is one of the major causes of early culling in sow operations worldwide and impacts animal welfare, productivity, and producer profitability. It is known that specific claw lesion types may be linked to increased predisposition to lameness (1, 2) and impaired reproductive performance (3, 4). Given the high prevalence of claw and foot lesions in slaughtered sows, which ranges from 88 to 100% (2, 5, 6), it is pivotal to understand the progression and severity of these disorders to develop effective nutritional and management strategies.

Some claw lesion types have been identified in sows and have different etiologies and pathogenesis. These include heel overgrowth and erosion (HOE), heel-sole crack (HSC), vertical (CVW) and horizontal (CHW) wall cracks, white line lesions (WL), overgrown toes (T), and dewclaws (DC). The most common disorder of the claw is HOE, mainly associated with standing/walking on hard surfaces, which increases the pressure on the side wall and white line, predisposing sows to lesions on those areas (2, 6, 7). Sows facing issues with HOE usually have various degrees of HSC, which is a consequence of constant tension, leading to fatigue on the tissue structure and rupture (8). Additionally, sows usually develop WL from HOE, as the white line is a fragile and flexible tissue that merges the elastic heel tissue and hard wall tissue (9). The WL is frequently aggravated by suboptimal flooring conditions, high humidity, and deteriorated sanitary conditions [i.e., poor cleaning or sanitation, decreased aeration rate, poor biosecurity precaution; (9)]. Lesions associated with HSC may compromise the corium, resulting in inflammation, infection, pain, and lameness (10). Cracks or fissures are common issues that can occur in a vertical (CVW; from the coronary band to the weight-bearing surface) or horizontal (CHW; parallel to the coronet) direction. The causes for CVW are less understood, and these lesions only develop into lameness when those cracks are deep (11). On the other hand, CHW are frequently associated with a physiological change leading to a disruption of hoof horn formation or a disease state, which interrupts horn formation in the hoof wall (11). Lastly, T and DC are linked with aging (11) and do not necessarily lead to lameness, mainly because of their subclinical and chronic nature (2, 12, 13). In severe cases, however, T and DC may become caught in slatted floors and may be completely ripped off. This can trigger the development of infections, lesions, and pain (14).

One of the major changes in Brazilian pork production in recent years has been technological advancements driven by increased exports. New arrangements in the pork supply chain, such as strictly coordinated systems, have shifted production structures from independent, smaller herds to larger and more advanced integrations and cooperatives (15). In Brazil, herds in the southern states of Paraná, Rio Grande do Sul, and Santa Catarina are particularly significant for integrators and cooperatives. This shift has led to improvements in nutrition, health status, and genetic material, which may collectively influence claw quality in commercial herds. In highly technified, integrated, or cooperative systems, herd management has considerable variation regarding maintaining breeds on-site (internal) or purchasing commercially available genetics (external). The approach taken may affect the development of claw lesions. Finally, it has been shown that WL is highly associated with lameness and is potentially more harmful in younger parity sows, while using group housing during gestation with electronic sow feeders predisposes sows to more severe lesions (16).

Recently, novel technologies, including mobile devices (i.e., for lesion recording), computer vision, and acoustic analyses, have been developed for claw lesion scoring and evaluation (17, 18). However, these technologies are still in the infancy of development, and many producers and field veterinarians still rely on visual evaluation of claws. Nonetheless, the processing and analyzing data from field observations is laborious as the number of animals and variables increases. Therefore, in veterinary science, multivariate statistical approaches have gained traction, allowing for testing the effects of factors on a smaller set of variables (19–21). There are several multivariate statistical approaches available including principal component analysis (PCA) and cluster analysis (hierarchical agglomerative cluster; HAC). Briefly, the PCA is often used to transform a larger set of variables into a smaller set that preserves most of the information in the large set (22). The HAC algorithm is a straightforward clustering technique that treats each data point as an individual cluster and then progressively agglomerates pairs of clusters until all clusters have been merged into a single cluster that contains all data (23).

Thus, this study assessed the status of claw lesions in commercial sow herds in Brazil by using the PCA and HAC approaches. These techniques were employed to identify the most important factors influencing claw health and describe their interactions. Moreover, partial least square (PLS) regression analysis was applied between the claw lesion traits on the classes defined by the cluster analysis.

## 2 Materials and methods

### 2.1 Animal care

The assessments were conducted on commercial herds in Brazil. All the procedures were reviewed and approved by the Ethics Commission of the Animal Ethics Committee (CEUA) from the Federal University of Paraná (Setor Palotina) under the Protocol number 16/2013-CEUA/Palotina. The assessments followed the appropriated guidelines of the Comissão Nacional de Bem-Estar Animal (COBEA). Furthermore, the members involved in animal assessment hold degrees in Veterinary Medicine and are experienced in evaluating claw lesion on live animals.

### 2.2 Herd selection criteria, lesion assessment, and database

**Figure 1:**
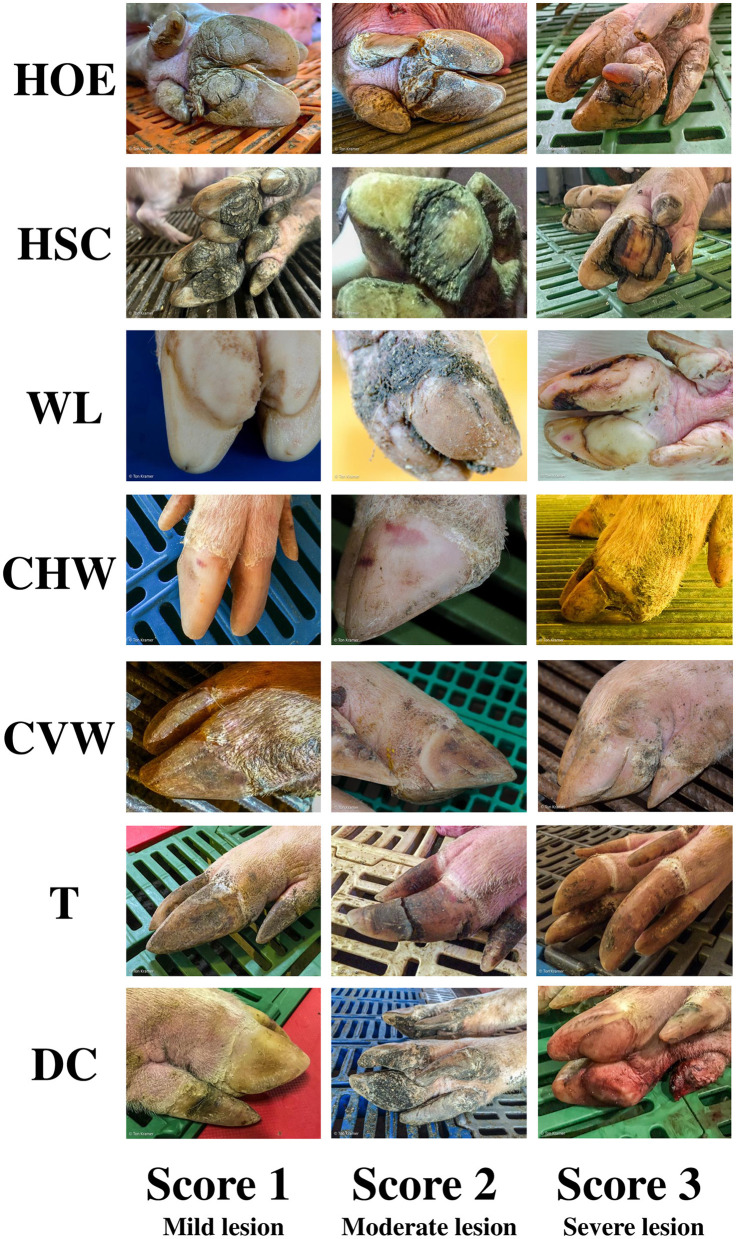
Sow claw lesion scoring system. Lesions: Heel overgrowth and erosion (HOE); Heel-sole crack (HSC); White line (WL); Horizontal (CHW) and vertical (CVW) wall cracks; Overgrown toes (T); Dewclaws (DC).

A total of 129 swine herds were assessed once by two independent experts for claw lesion severity from 2013 to 2023. After parturition, sows (*n* = 12,364; ≥10% of the inventory size within each heard, up to a maximum of 100 animals) were randomly selected and scored utilizing a scale from 0 (no lesions) to 3 (severe lesions) for claw lesions in the hind legs (2) by one evaluator. Lesions included HOE, HSC, WL, CHW, CVW, T, and DC. A lesion index (L-Index) was calculated as the sum of the higher scores from each lesion per sow. Figure 1 shows the lesion scoring system used according to lesion type and severity. The following criteria were used to include data from a given herd in the database: herds employing similar management protocols in terms of sow movement, management, and feeding; herds with similar housing conditions during the lactation phase; sows were only included in the assessment if they (1) were not used as foster sows in previous parities, (2) had good health status (e.g., no prolapse, reproductive disorder, or abortion in previous parities), (3) had acceptable body condition score between 2.5 and 3.0 [1.0–5.0 scale; Young et al. (24)].

**Figure 2:**
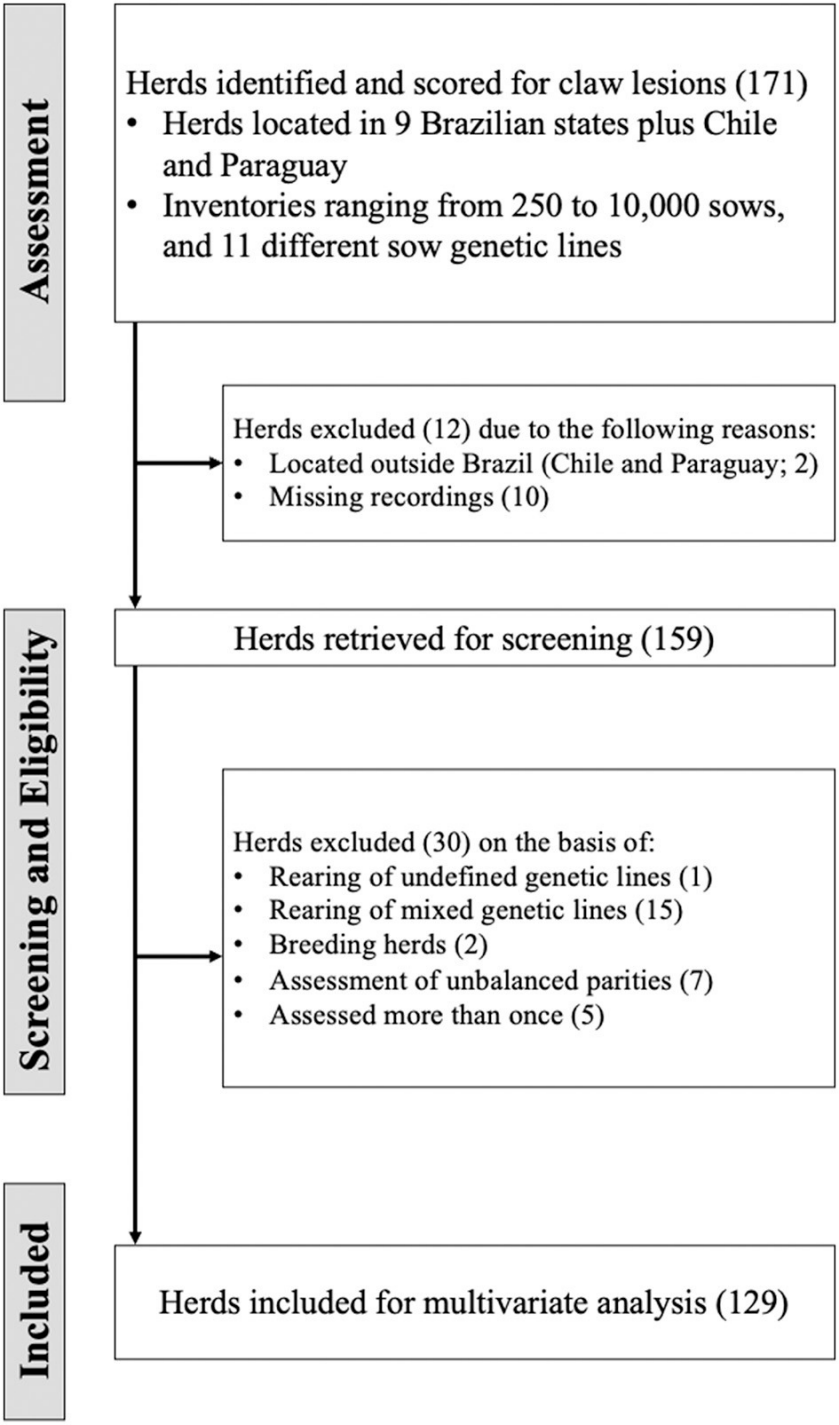
Flow of information through the different phases of the assessment, screening, and eligibility procedure of herds.

Figure 2 represents an illustrative diagram of the workflow of eligible herd selection for this study. Commercial herds located in nine Brazilian states [Distrito Federal (2), Goiás (23), Minas Gerais (3), Mato Grosso (1), Mato Grosso do Sul (12), Paraná (77), Rio Grande do Sul (10), Santa Catarina (32), São Paulo (8)], Chile (1), and Paraguay (1)] were assessed for claw lesions according to the methodology described above. The initial selection included a total of 26,030 sows evaluated, with an average of 102 ± 0.04 sows evaluated per herd. The average inventory size of herds was 2,275 sows (ranging from 250 to 10,000 sows; mode: 500). After the assessment, relevant herds were critically evaluated in terms of fitness to the objectives of the present multivariate analysis approach. Since one of the main purposes of the present study was to identify patterns of claw lesion responses in Brazilian sow herds, the herds in Chile and the herd in Paraguay were excluded. Furthermore, it was determined that 10 herds had incomplete records on the number of sows assessed. Therefore, incomplete records was used as a second selection criterion, and resulted in the exclusion of an additional 10 herds. Subsequently, a checklist was performed in the selected herds to define their inclusion in the meta-analysis. The main criteria for herd exclusion were: (a) herds rearing undefined genetic lines, which consisted basically of rudimentary lines (*n* = 10), (b) herds where mixed genetic lines were raised (e.g., PIC and DanBred within the same herd; *n* = 15), (c) an unbalanced number of parities was assessed (*n* = 7), (d) breeding herds (*n* = 2), (e) herds evaluated more than once (*n* = 5). The final database ended up with 129 herds (*n* = 12,364 sows) for this study. After performing the screening procedure, the information relative to the proposed model, which is discussed in detail below, and outcome variables (i.e., HOE, HSC, WL, CHW, CVW, T, DC, and L-Index) were tabulated using a database from an electronic data spreadsheet.

### 2.3 Characterization of the database

Table 1 shows each specific lesion's average, median, mode, and SD within a year. Table 2 shows the number of sows and herds assessed per year according to the factors extracted for analysis, which are explained in detail below.

**Table 1:** Average, median, mode, and standard deviation (SD) of claw lesions collected by the two independent evaluators from 2013 to 2023.^a^

**Year**	**2013**	**2014**	**2015**	**2016**	**2017**	**2018**	**2019**	**2020**	**2021**	**2022**	**2023**
**Heel overgrowth and erosion**											
Average	1.61	1.37	1.13	1.19	0.96	0.45	1.00	0.84	1.09	0.85	0.84
Median	2	2	1	1	1	0	1	1	1	1	1
Mode	2	2	1	1	1	0	1	0	2	1	0
SD	0.82	0.81	0.77	0.84	0.85	0.74	0.74	0.88	0.90	0.82	0.83
**Heel-sole crack**											
Average	0.27	0.10	0.15	0.27	0.40	0.14	0.09	0.14	0.17	0.13	0.05
Median	0	0	0	0	0	0	0	0	0	0	0
Mode	0	0	0	0	0	0	0	0	0	0	0
SD	0.67	0.43	0.57	0.73	0.81	0.55	0.45	0.54	0.63	0.53	0.32
**White line**											
Average	0.99	0.55	1.04	1.16	1.14	0.73	1.31	1.22	1.58	1.50	0.92
Median	0	0	0	0	0	0	1	1	2	1	0
Mode	0	0	0	0	0	0	0	0	3	3	0
SD	1.15	0.94	1.19	1.28	1.28	1.12	1.30	1.30	1.39	1.18	1.08
**Horizontal wall crack**											
Average	1.08	0.75	1.19	1.09	0.81	0.36	1.08	1.03	0.93	0.44	0.33
Median	1	1	1	1	1	0	1	1	1	0	0
Mode	1	1	1	1	1	0	1	1	1	0	0
SD	0.79	0.75	0.70	0.75	0.79	0.85	0.88	0.80	0.85	0.82	0.73
**Vertical wall crack**											
Average	0.49	0.47	0.43	0.43	0.32	0.17	0.41	0.24	0.26	0.21	0.19
Median	0	0	0	0	0	0	0	0	0	0	0
Mode	0	0	0	0	0	0	0	0	0	0	0
SD	0.85	0.81	0.82	0.84	0.75	0.56	0.87	0.69	0.73	0.57	0.54
**Overgrown toes**											
Average	1.01	0.68	0.48	0.43	0.41	0.26	0.22	0.17	0.13	0.10	0.06
Median	1	1	0	0	0	0	0	0	0	0	0
Mode	1	1	0	0	0	0	0	0	0	0	0
SD	0.61	0.66	0.60	0.73	0.71	0.57	0.54	0.50	0.41	0.39	0.29
**Dewclaws**											
Average	1.20	1.01	0.39	0.64	0.83	0.92	0.90	0.80	0.33	0.34	0.19
Median	1	1	0	0	1	1	1	0	0	0	0
Mode	2	0	0	0	0	0	0	0	0	0	0
SD	0.87	0.91	0.71	0.85	0.85	0.88	0.94	0.91	0.63	0.68	0.52
**Lesion index**											
Average	6.66	4.92	4.81	5.22	4.87	3.03	5.01	4.45	4.49	3.57	2.58
Median	6	5	4	5	5	2	5	4	4	3	2
Mode	6	4	3	4	6	0	3	4	3	3	2
SD	3.25	3.05	2.75	2.83	3.22	2.91	2.83	2.88	2.78	2.43	2.10

^a^Within each farm, one randomly selected group of sows was scored by the same evaluator from 0 (none) to 3 (severe) for heel overgrowth and erosion (HOE), heel-sole crack (HSC), separation along the white line (WL), horizontal (CHW) and vertical (CVW) wall cracks, and overgrown toes (T), or dewclaws (DC) in the hind legs after parturition.

**Table 2:** Records of claw lesions collected by the two independent evaluators from 2013 to 2023, the number of herds, sows, and distribution of the recorded population on parity groups, region, months, inventory sizes, breeds, and gestation housing type.^a^

**Year**	**2013**	**2014**	**2015**	**2016**	**2017**	**2018**	**2019**	**2020**	**2021**	**2022**	**2023**
Sow herds assessed, *n*	16	7	12	20	3	3	3	7	5	29	24
Sows assessed, *n*	1,558	856	1,096	1,732	298	306	306	734	436	2,627	2,415
**Sows and (herds) assessed by production structure** ^b^											
Coop	396 (4)	505 (4)	99 (1)	575 (5)	0 (0)	205 (2)	0 (0)	321 (3)	183 (2)	1,479 (18)	497 (6)
Integrator	43 (1)	0 (0)	372 (5)	1,055 (14)	199 (2)	101 (1)	100 (1)	105 (1)	0 (0)	829 (8)	1,767 (16)
Independent	1,119 (11)	351 (3)	625 (6)	102 (1)	99 (1)	0 (0)	206 (2)	308 (3)	253 (3)	319 (3)	151 (2)
**Sows assessed by parity**											
Parity 1	343	204	359	536	48	80	129	214	260	673	488
Parity 2	271	139	176	428	58	75	44	97	27	488	422
Parity 3	319	105	165	228	66	98	56	96	37	459	399
Parity 4	176	100	113	157	44	13	22	93	30	380	338
Parity 5	152	81	86	131	31	30	21	73	30	199	283
Parity 6	87	75	68	102	22	2	10	88	14	180	215
Parity 7	210	152	129	150	29	8	24	73	38	248	270
**Sows and (herds) assessed by region** ^c^											
South	1,345 (11)	505 (3)	523 (7)	1,527 (12)	298 (3)	306 (3)	206 (2)	734 (0)	436 (0)	1,798 (21)	648 (8)
Southeast	213 (4)	158 (2)	0 (0)	102 (1)	0 (0)	0 (0)	0 (0)	0 (0)	0 (0)	0 (0)	0 (0)
Midwest	0 (0)	193 (2)	573 (5)	103 (1)	0 (0)	0 (0)	100 (1)	0 (0)	0 (0)	829 (8)	1,767 (16)
**Sows and (herds) assessed by inventory sizes**											
250 to 810 sows	273 (5)	58 (1)	300 (4)	811 (12)	0 (0)	0 (0)	118 (1)	104 (1)	56 (1)	413 (8)	174 (3)
810 to 1,300 sows	848 (6)	0 (0)	188 (2)	303 (3)	190 (2)	0 (0)	188 (2)	206 (2)	275 (3)	967 (9)	1,101 (11)
1,300 to 3,000 sows	138 (2)	293 (3)	305 (3)	101 (1)	108 (1)	204 (2)	0 (0)	207 (2)	105 (1)	630 (6)	1,140 (10)
3,000 to 10,000 sows	299 (3)	505 (4)	303 (3)	517 (4)	0 (0)	102 (1)	0 (0)	217 (2)	0 (0)	617 (6)	0 (0)
**Sows and (herds) assessed by breed type** ^d^											
Internal	0 (0)	0 (0)	320 (4)	954 (13)	0 (0)	0 (0)	100 (1)	105 (1)	156 (2)	104 (1)	691 (7)
External	1,558 (16)	856 (7)	776 (8)	778 (7)	298 (3)	306 (3)	206 (2)	629 (6)	280 (3)	2,523 (28)	1,724 (17)
**Sows and (herds) assessed by gestation housing type** ^e^											
Individual	1,558 (16)	663 (6)	1,096 (12)	1,732 (20)	298 (3)	306 (3)	206 (2)	522 (6)	436 (7)	1,185 (14)	569 (7)
Group	0 (0)	193 (1)	0 (0)	0 (0)	0 (0)	0 (0)	100 (1)	112 (1)	0 (0)	1,442 (15)	1,846 (17)

^a^For production structure, parity, region, inventory sizes, breed and housing type, values are number of sows assessed and values between parenthesis are number of farms assessed.

^b^Herds belonged to three different production structures, namely Coop (41), Integrator (50), and Independent (38). The Coop provides the pigs and feed to Coop members at the cost of production. Profits from the Coop after slaughtering and marketing the pork apportioned back to producers based upon the total number of pigs marketed. In the Integrator structure, the largest company (integrator) offers pigs, feed, and technical assistance to the farmer, and is responsible for slaughtering and commercialization of pork. The farmer provides facilities, equipment, heating, water, and labor (generally family labor). At slaughter age, pigs are retrieved from farmers by the Integrator. In the Independent structure, the farmer make their own decisions on pig and feed sourcing, quality assurance, sanitary management, and commercialization. These are usually smaller herds.

^c^Herds were in the Midwest (n = 34), Southeast (n = 7), or South (n = 88) regions of Brazil. Tropical Savanna, Monsoon-influenced Humid Subtropical, and Humid Subtropical climates, respectively, characterize these regions.

^d^Management of the herd was conducted either as a closed herd genetic approach, where breeds were manintained from stock on-site (internal), or through purchase of commercially available genetics (external). The latter including purchased sows, boars, or semen through breeders, live auctions, or boar studs.

^e^Herds adopted either individual crates (n = 88 herds) or group housing (n = 41 herds) during gestation. The individual housing system was fairly consistent across herds, providing ~2.40 × 0.65 m (length × width, 1.56 m^2^/head) with an individual feeder and drinker. The individual housing was slightly larger than the size of the sow's body. In this sense, there was only enough room for the sow to stand or lie down in place, with no room for the sow to turn around or move freely. The gestating sows of the herds adopting group housing system were housed in rooms with approximate measures of 10.5 × 14.4 m (5.04 m^2^/head). Sows in the group house could move freely. All the herds had similar farrowing units equipped with individual farrowing crates. Each crate measured ~1.10 × 2.41 m, with a piglet area of ~1.53 × 0.53 m, which had an accessible creep area, rubber mats, and a heat lamp. Individual bowl feeders and nipple drinkers were located at the front of each sow space.

Herds were in the Midwest (*n* = 34), Southeast (*n* = 7), or South (*n* = 88) regions of Brazil. Tropical Savanna, Monsoon-influenced Humid Subtropical, and Humid Subtropical climates, respectively, characterize these regions. Inventory sizes ranged from 250 to 10,000 sows (2,907.55 ± 2,352.06). Inventory sizes were further stratified into 250–810, 811–1,300, 1,301–3,000, and 3,001–10,000 sows. Herds belonged to three different production structures, namely Cooperative (Coop; 41), Integrator (50), and Independent Producers (38). The Coop provides the pigs and feed to Coop members at the cost of production. After slaughtering and marketing the pork, profits from the Coop are apportioned back to producers based on the total number of pigs marketed. In the Integrator structure, the largest company (integrator) offers the farmer pigs, feed, and technical assistance and is responsible for slaughtering and commercializing the pigs. The farmer provides facilities, equipment, heating, water, and labor. At slaughter age, pigs are retrieved from farmers by the Integrator. In the Independent structure, the farmer makes their own decisions on pig and feed sourcing, quality assurance, sanitary management, and commercialization. These are usually smaller herds. Management of the herd was conducted either as a closed herd genetic approach, where breeds were maintained from stock on-site (internal), or through purchase of commercially available genetics (external). The latter including purchased sows, boars, or semen through breeders, live auctions, or boar studs. Sows were housed in individual crates (n = 88 herds) or group housing (*n* = 41 herds) during gestation. The individual housing system was fairly consistent across herds, providing ~2.40 × 0.65 m (length × width, 1.56 m^2^/head) of space per sow with an individual feeder and drinker in the crate. The individual housing was slightly larger than the size of the sow's body. In this sense, there was only enough room for the sow to stand or lie down in place, with no room for the sow to turn around or move freely. The gestating sows in the group housing systems were housed in pens measuring 10.5 × 14.4 m (5.04 m^2^/head). Sows in the group house could move freely. All the herds had similar farrowing units equipped with individual farrowing crates. Each crate measured ~1.10 × 2.41 m, with a piglet area of ~1.53 × 0.53 m, which had an accessible creep area, rubber mats, and a heat lamp. Individual bowl feeders and nipple drinkers were located at the front of each sow space.

### 2.4 Variable definition and encoding

The methodology described by Sauvant et al. (25) and Lovatto et al. (26) was used in the present study for the definition of dependent and independent variables and data encoding. The encodings were used as qualitative and quantitative variables in the analysis with the purpose of considering the herd, inter and intra variability of the compiled data. Specific sequential numbers were used for each herd inserted in the database to encode the herd effect. Within each herd, each sow received sequential numbers for labeling, which characterizes the inter-encoding. This allowed to assign the sow encoding with the specific herd encoding. The intra-encoding, following a similar pattern as described above, was attributed to each factor within sows and herds. The intra encoding was composed by the following: (1) Production structure (Coop, Integrator, Independent); (2) Region (South, Southeast, Midwest); (3) Inventory size (250–810, 811–1,300, 1,301–3,000, and 3,001–10,000 sows); (4) Breed (internal, external); (5) Gestation housing (individual, group); and (6) Parity (1–7). Design criteria included Production structure, Region, Inventory size, Breed, Gestation housing, and parity. The dependent variables extracted for analysis were HOE, HSC, CVW, CHW, WL, T, DC, and L-Index.

### 2.5 Statistical analysis

Data were analyzed using a multivariate approach whereby relationships between claw lesion variables (HOE, HSC, WL, CHW, CVW, T, and DC) were studied by PCA and HAC. The data were analyzed using SAS, version 9.4 (SAS Institute Inc., Cary, NC, USA) and XLSTAT2017.19.6 (AddinSoft, Paris, France). Data normality was checked based on visual inspection of the raw data histogram and QQ-plots. When variables were not considered normally distributed, categorical variables were encoded as dummy variables, to allow for them to be used as predictors in the PLS approach. For the Cluster analysis, the dissimilarity and similarity measures were tailored to categorial data, which does not require for any type of data transformation.

**Figure 3:**
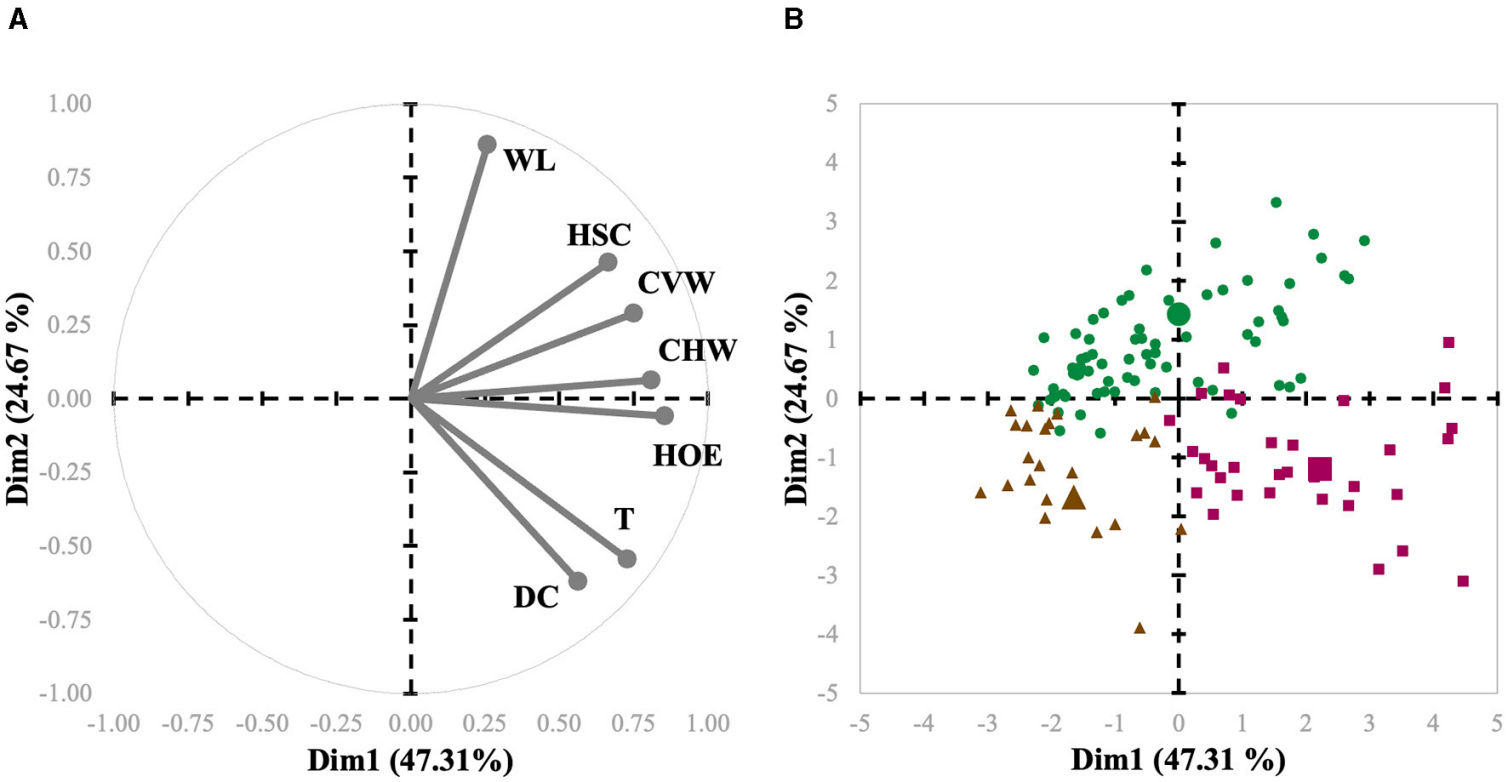
Biplot of variables **(A)** and herds **(B)** used in the meta-analysis of sows represented for the first (Dim1) and second dimensions (Dim2). Each herd is represented by symbols, and their shapes indicate to which cluster they belong (Cluster 1 = green circles; Cluster 2 = purple squares; Cluster 3 = brown triangles). The cluster centroid is represented by a bigger symbol with the same shape as the herds in the same cluster.

The main objective of the present study was to implement PLS based on clustering methods; thus, herds were first categorized in accordance with their individual claw lesion scores. The claw lesion scores were averaged per herd prior to any clustering analysis and subsequently used for clustering using HAC. To achieve this, the PROC CLUSTER procedure of SAS was used. This analysis is a task of exploratory data mining, and our main objective was grouping (clustering) the data set in such a way that the herds in the same group (cluster) were more similar to each other than to those in other groups (clusters). First, the function builds a hierarchical tree; then, the sum of the within-cluster inertia is calculated for each partition. Thereby, the suggested partition is the one with the higher relative loss of inertia. The herds were grouped according to similarity in three clusters, i.e., C1, C2, and C3. The three clusters were projected on the graph defined by the principal components (PC1 and PC2; Figure 3). The number of clusters was supported by practical and biological interpretation.

PLS regression (27) was used to examine claw lesion data on the three clusters defined by the HAC. In this stage, the number of components is automatically determined using the Q2 statistic, assessing the significance of a new PLS component in predicting the entire Y set. PLS projects the predictive and observed variables into a new space by finding pairs of weight vectors that maximize the covariance between the two projections. By examining this new space, the underlying relationship between clusters and claw lesions can be explored (28). Subsequently, confidence intervals for PLS regression coefficients (bkj) are calculated through jackknife. The contribution of each factor to the model in terms of the variance explained was indicated by the variable importance in projection (VIP) and the standardized regression coefficients were estimated as well to confirm the selection of the variables. In this sense, a VIP ≥ 1 was assumed as a selection threshold. To achieve this, the PROC PLS procedure of SAS was used.

## 3 Results and discussion

Scoring claw lesions is a labor- and time-intensive process, and clinical lameness is only visually apparent when a significant claw lesion has occurred. Despite the recent development of novel technologies to detect claw lesions (i.e., acoustic analysis, infrared thermography, sensors), these are still in the infancy stages of development and may be cost-prohibitive (18, 29, 30). Therefore, many producers, veterinarians, and extension experts still rely on visual, on-farm claw assessment. Since visual assessment is subjective, supportive strategies must be adopted to handle and analyze data coming from claw quality scoring, particularly as the number of animals/herds evaluated increases. The integration of HCA, PCA, and PLS methods in this study offers a valuable tool for the swine industry, enabling the identification and exploration of significant variables within vast and interconnected production datasets. Additionally, clustering techniques prove beneficial for decision-making in an industry constrained by the limitations of daily monitoring of numerous datasets.

### 3.1 Phenotypic correlations

**Figure 4:**
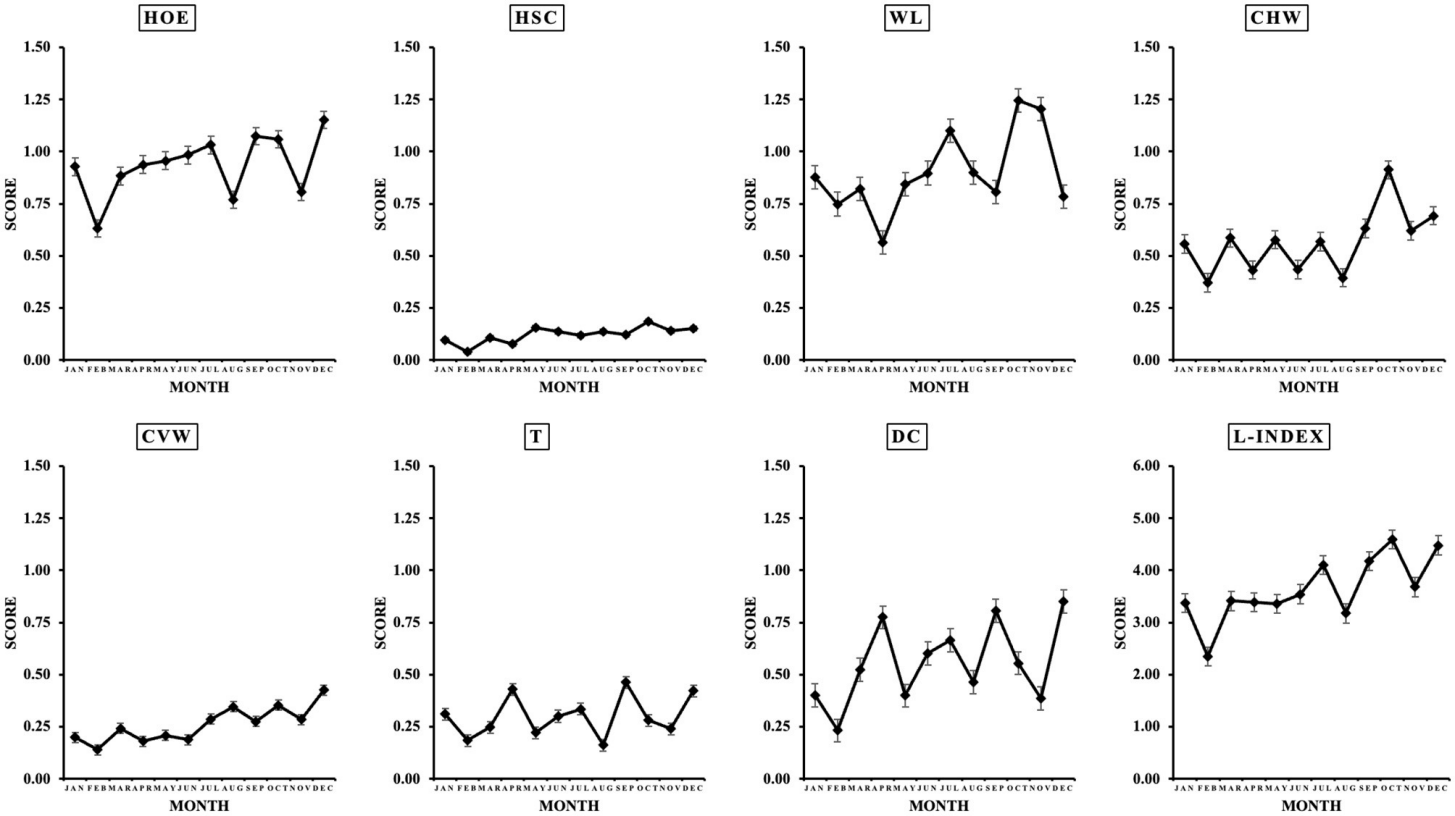
Monthly variation in claw lesion [heel overgrowth and erosion (HOE), heel-sole crack (HSC), white line (WL), horizontal (CHW) and vertical (CVW) wall cracks, overgrown toes (T), and dewclaws (DC)] score. Values are average ± SEM.

Table 1 shows the average, median, and mode values for each lesion per evaluation year. Heel overgrowth and erosion average score was 1.05 ± 0.38, ranging from 0 to 3, with mode 1. Heel-sole crack average score was 0.16 ± 0.15, ranging from 0 to 3, with mode 0. White line average score was 1.18 ± 0.57, ranging from 0 to 3, with mode 1. Horizontal wall cracks average score was 0.73 ± 0.45, ranging from 0 to 3, with mode 0. Vertical wall cracks average score was 0.32 ± 0.22, ranging from 0 to 3, with mode 0. Overgrown toes average score was 0.33 ± 0.37, ranging from 0 to 3, with mode 0. Dewclaws average score was 0.55 ± 0.47, ranging from 0 to 3, with mode 0. It is important to highlight that herds were assessed at different time points throughout the year since this was a long-term evaluation project. Since the month of evaluation did not have an effect on the severity of any of the claw lesions (Figure 4), it was not included in the model.

Table 2 summarizes the number of sows and herds assessed per year stratified according to the factors examined (i.e., production structure, parity, inventory size, etc.). The number of assessments per factor was reasonably consistent throughout the years, except for breed and gestation housing types. Parity was equalized as much as possible within sample time, ranging from 1 to 7 in all of the sample times. The number of sows and farms (in parenthesis) assessed for internal and external breeds was 2,430 (29) and 9,934 (100), respectively, and 8,571 (96) and 3,793 (33) for individual and group gestation housing, respectively. These are expected, as the breeding herd component may represent ~20% of the total feed produced and can be cost-prohibitive for many production systems (31). In this sense, purchasing the breeding stock from external sources is generally more advantageous. Recently, Klein et al. (32) reported that gilt replacement protocol may impact sow farms' claw lesions. Regarding gestation group housing, the Brazilian Ministry of Agriculture, Livestock and Food Supply recently released the first pig welfare legislation (Normative Instruction # 211, February 1, 2021), which establishes that group housing during gestation is mandatory and sows should not be kept in individual crates for more than 35 days of gestation. This is also consistent with findings from the present study, with an increase in the percentage of herds using group housing in 2022 (52% of herds evaluated) and 2023 (71% of herds evaluated) compared to the previous year (1% of herds evaluated; 2013–2021).

Heel overgrowth and erosion had the highest prevalence throughout the years, followed by WL and CHW. This is consistent with multiple reports in the literature showing that the most frequently observed claw lesions in sows are HOE, WL, and CHW (6, 9). Furthermore, the lowest scores were observed for T, DC, and CVW, which is similar to the findings from Henningsen (33) in a retrospective study on U.S. sow farms. The authors observed that toe abnormalities were not as severe as previously believed and that histopathology did not indicate a direct relationship between these abnormalities and weight-bearing. This was due to the fact that similar lesions were present in both the lateral toes and lateral dew claws.

Interestingly, L-Index had a progressive decrease from 2013 to 2023 (Table 1). This finding may be due to a combination of factors. First, it is important to consider that claw size and hoof growth have gained traction as selection criteria due to their important genetic background, directly impacting sow culling (4). Secondly, despite being speculative, improvements in housing (flooring) conditions have certainly been made throughout the years, reducing the predisposition of sows to claw lesions (34). Also, refinements in feeding practices during the past 10 years may have been allowed for a better support of claw health in these herds. For example, nutrient requirements for gestating and lactating sows were solely based on growth and reproductive performance for many years, and advancements in mineral and vitamin nutrition have shown that they play a pivotal role in maintaining optimum foot health in sows (35, 36). The herds were grouped in three clusters, i.e., C1, C2, and C3. From the multivariate perspective, the spatial distribution of each observation related to the first and second principal components and variable axis are presented as a biplot in Figures 3A, B, representing a condensed summary of the database structure. These two components represented a cumulative inertia of 71.98%. Figure 3A also shows that HOE, CHW, and CVW contributed most to the first component, with coordinates of 0.86, 0.81, and 0.75, respectively, while WL, DC, and T contributed most to the second component, with coordinates of 0.86, −0.62, and −0.55, respectively. It is also possible to compare clusters regarding claw lesion variables by observing their centroid position in these 2 dimensions. Hence, it is possible to conclude that C3, compared to the other clusters, is represented by herds with lower scores for all the lesions except for DC. It is also possible to evaluate variable relationships since the angle between their axes reveals how they relate. The smaller the angle between the 2 variables, the more positively correlated they are, which may be concluded from the relation between T and DC, and HOE and CHW. Indeed, HOE is one of the most prominent lesions affecting the outer claw of the hind leg due to weight-bearing biomechanics (9, 37). The softer heel horn, comprising fewer tubules, bears most of the weight (38), unlike other cloven-hoofed animals where the sole is predominant. Since the occurrence of HOE is highly associated with flooring surface abrasiveness (39, 40), it is common to observe its cooccurrence with wall cracks (9), which is in line with our findings. Moreover, and on the contrary, variables with opposite directions are negatively correlated, which means that for this database, the higher the WL score for a given sow is, the lower the score for T and DC may be. This also makes biological sense, as T and DC are lesions particularly observed in older sows (10), while WL is generally present earlier and leads to sow culling in many situations (41–43).

### 3.2 Multivariate approach

In a univariate approach, C3 herds were characterized by having lower scores for all the lesions compared to C1 and 2, except for DC, which were basically the same across clusters (Table 3). Specifically, HOE, HSC, WL, CHW, CVW, and T were decreased by 17, 25, 11, 25, 21, and 17%, respectively, in C3 compared to C1 and 2 combined. In comparing C1 and C2, they had similar scores for individual lesions. The L-Index was greater in C2 compared to C1, which indicates that overall claw quality was further deteriorated in C2 herds despite C1 being affected by multiple lesions.

**Table 3:** Descriptive statistics of claw lesion traits assessed in different sow herds used in the multivariate approach split by cluster groups^a^.

**Item^b^**	**Cluster 1**	**Cluster 2**	**Cluster 3**
HOE	1.09 ± 0.05	1.07 ± 0.07	0.90 ± 0.06
HSC	0.17 ± 0.02	0.15 ± 0.03	0.12 ± 0.02
WL	1.22 ± 0.07	1.16 ± 0.10	1.06 ± 0.11
CHW	0.76 ± 0.05	0.77 ± 0.09	0.58 ± 0.08
CVW	0.34 ± 0.05	0.32 ± 0.09	0.26 ± 0.08
T	0.32 ± 0.04	0.37 ± 0.07	0.29 ± 0.07
DC	0.54 ± 0.06	0.55 ± 0.08	0.56 ± 0.09
L-Index	4.07 ± 0.17	5.03 ± 0.31	4.01 ± 0.38

^a^HOE, heel overgrowth and erosion; HSC, heel-sole crack; WL, white line; CHW, horizontal wall crack; CVW, vertical wall crack; T, overgrown toes; DC, dew claws; L-Index, Lesion index.

^b^Data are expressed as average score within cluster ± SEM.

Table 4 shows the characterization of the herds according to cluster group. There was an increased proportion of herds and sows belonging to C1 (56% of herds) compared to C2 (26% of herds) and C3 (18%). This remarkable finding shows that most sows may be at risk in terms of claw quality, and sows may indeed be suffering from multiple lesions. Regarding production, the three clusters followed the same trend, with a higher proportion of herds in Coop and Integrator structures than in Independent. This exemplifies a transition seen in the Brazilian swine industry, with more producers shifting to Integrator or Coop structures. This is mainly driven by price and market, which are absorbed by Integrators and diluted in Coop environments. Most herds from C1 and C2 were present in the South region, followed by the Midwest, with only a few located in the Southeast. On the other hand, C3 had almost the same number of herds located in the South and Midwest, with a few herds in the Southeast. The South and Midwest regions represent the main pork production regions in Brazil, geographically positioned closer to grains, grow/finishing facilities, and packing plants. C1 herds were homogeneously distributed across inventory sizes. C2 had the majority of herds with inventories ranging from 810 to 1,300 and no herds with more than 3,000 sows. C3 was basically characterized by a lower percentage of herds with inventory sizes of 250–810 sows. Breed type did not differ among clusters, with a higher proportion of external than internal breeds. The proportion of herds with group gestation housing increased progressively from C1 through C3.

**Table 4:** Characterization of cluster groups according to the factors examined.

**Item**	**Cluster 1**	**Cluster 2**	**Cluster 3**
Number of herds and (sows)	72 (6,886)	33 (3,002)	24 (2,476)
**Sows by production structure**			
Coop	2,223 (32%)	1,176 (39%)	861 (35%)
Integrator	2,672 (39%)	1,004 (33%)	895 (36%)
Independent	1,991 (29%)	822 (28%)	720 (29%)
**Sows by region**			
South	4,833 (70%)	2,203 (73%)	1,290 (52%)
Southeast	219 (3%)	186 (6%)	68 (3%)
Midwest	1,834 (27%)	613 (21%)	1,118 (45%)
**Sows by inventory sizes**			
250 to 810 sows	1,535 (22%)	498 (17%)	274 (11%)
810 to 1,300 sows	2,061 (30%)	1,646 (55%)	559 (23%)
1,300 to 3,000 sows	1,453 (21%)	858 (29%)	920 (37%)
3,000 to 10,000 sows	1,837 (27%)	0 (0%)	723 (29%)
**Sows by breed type**			
Internal	1,257 (18%)	693 (23%)	480 (19%)
External	5,629 (82%)	2,309 (77%)	1,996 (81%)
**Sows by gestation housing type**			
Individual	4,995 (73%)	1,922 (64%)	1,450 (59%)
Group	1,891 (27%)	1,080 (36%)	1,026 (41%)

**Figure 5:**
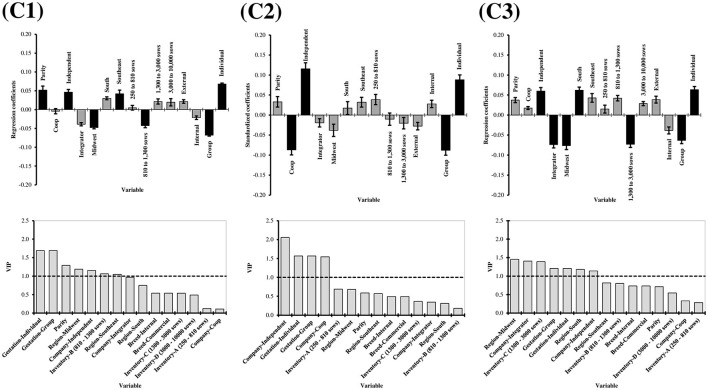
Partial least square (PLS) coefficients and variable importance in projection (VIP) for lesion index of claw lesion clusters (C1 = Cluster 1, C2 = Cluster 2, C3 = Cluster 3). For the regression coefficients charts, the bars indicate 95% confidence intervals based on jackknifing. Moreover, black bars represent factors with VIP equal to or above 1. Factors: Parity (1–7); Production structure (Coop, Integrator, and Independent); Region (South, Southeast, and Midwest); Inventory size (250–810, 810–1,300, 1,300–3,000, and 3,000–10,000 sows); Breed (internal, external); Gestation housing type (group, individual).

The selection of variables in PLS regression models depends on defined thresholds, such as VIP, which was chosen in the present study (44). However, these thresholds are not strict limitations, and variables near them should also be considered (45), especially given the multifactorial nature of claw quality (8, 17, 46). Variable selection was conducted separately for each cluster to enhance generalization to other herds, favoring a more generalized approach (Figure 5). The L-Index within each cluster was used as the response variable to employ the PLS regression. This was done to investigate whether the factors leading to overall claw quality deterioration would differ between clusters.

The L-Index increased with increasing parity (VIP = 1.291, regression coefficient = 0.052), which was expected from the aging effect on claw quality previously discussed (Figure 5). However, this was observed in C1 only. This suggests that parity increase does not always lead to deterioration in claw quality and confirms the multifactorial scenario by which claw lesions are influenced. Independent production structure increased the L-Index in all three clusters (C1: VIP = 1.154, regression coefficient = 0.046; C2 = VIP = 2.056, regression coefficient = 0.116; C3: VIP = 1.140, regression coefficient = 0.060). This was concomitant with Coop and Integrator production structures, reducing the L-Index in C2 (VIP = 1.544, regression coefficient = −0.087) and 3 (VIP = 1.406, regression coefficient = −0.074), respectively. Better claw quality in Integrator/Coop structures compared to Independent may be explained by a higher standardization of processes, including genetic material, nutrition, technical expertise, and facilities. The Southeast and South regions had increased L-Index in C1 (VIP = 1.046, regression coefficient = 0.042) and 2 (VIP = 1.181, regression coefficient = 0.062), respectively, while the Midwest region had decreased L-Index in C2 (VIP = 1.189, regression coefficient = −0.048) and C3 (VIP = 1.453, regression coefficient = −0.076). It is important to highlight the higher proportion of herds in the Midwest in C3 (45%) compared to C1 and C2 (around 21%). This may also be explained by a recent trend in Brazilian herds, with the South directing more pork to exports and the Midwest raising a new, technified, integrated source of pork to meet internal demands.

Individual housing increased the L-Index regardless of cluster (C1: 1.694, regression coefficient = 0.068; C2: VIP = 1.567, regression coefficient = 0.088; C3: VIP = 1.206, regression coefficient = 0.063). This goes against findings from Anil (2), who conducted claw lesions scoring in 184 sows in farrowing stalls on day 110 of gestation, associating them with gestation housing system (individual vs. group), using logistic regression models. The authors reported that the prevalence of claw lesions among gestating sows was significantly higher in group pens with electronic sow feeding (ESF) compared to stalls, except for toe lesions. The majority of sows with various types of claw lesions, including wall, heel, white line, heel-sole junction, sole, and overgrown heels, were housed in group pens during gestation, ranging from 57.8 to 75.4%. This increased occurrence of claw lesions in sows housed in group pens with ESF systems could be attributed to fighting and aggressive behavior during mixing and feeding times. The elevated mobility afforded by the ESF system may have contributed to this discrepancy compared to sows housed in stalls.

## 4 Conclusions

In the present multivariate approach, differences in claw lesions and their correlations among six types of claw lesions in sows housed under commercial conditions in Brazil from 2013 to 2023 were examined. The most prevalent lesions were HOE, WL, and CHW. Clustering techniques revealed that some herds were affected by multiple lesions, including HSC, WL, CHW, CVW, and T. The study also highlighted the influence of gestation housing, parity, and production factors on sow claw health. Sows in group gestation housing had a lower L-Index compared to those in individual housing, which merits further validation, as Brazil has only recently transitioned from individual housing practices. Moreover, independent production structures increased the L-Index, indicating poorer overall claw quality. Parity increase led to claw quality deterioration in a specific group of farms (C1), suggesting that claw lesions are influenced by a multifactorial scenario.

## Data availability statement

The original contributions presented in the study are included in the article/supplementary material, further inquiries can be directed to the corresponding author.

## Ethics statement

The animal study was approved by Erica Cristina Bueno do Prado Guirro, UFPR/Brazil. The study was conducted in accordance with the local legislation and institutional requirements.

## Author contributions

TK: Conceptualization, Funding acquisition, Investigation, Methodology, Project administration, Resources, Validation, Visualization, Writing – original draft, Writing – review & editing. AC: Validation, Visualization, Writing – review & editing. AK: Data curation, Investigation, Methodology, Validation, Writing – review & editing. MS: Investigation, Methodology, Validation, Writing – review & editing. CR: Writing – review & editing. LR: Conceptualization, Data curation, Formal analysis, Investigation, Methodology, Software, Validation, Visualization, Writing – original draft, Writing – review & editing. GA: Conceptualization, Data curation, Formal analysis, Funding acquisition, Investigation, Methodology, Project administration, Resources, Supervision, Validation, Visualization, Writing – original draft, Writing – review & editing.

## References

[1] Gjein H Larssen RB. The effect of claw lesions and claw infections on lameness in loose housing of pregnant sows. Acta Vet Scand. (1995) 36:451–9. 10.1186/BF03547660 8669373 PMC8095415

[2] Anil SS. Factors associated with claw lesions in gestating sows. J Swine Health Prod. (2007) 15:78–83.

[3] Wilson ME Ward TL. Impact of lameness on productive potential of the sow. In: 12th Lond Swine Conf Proc Time Change Lond Ont Can 28–29 March 2012. Ontario (2012). p. 27–33.

[4] Pluym LM Van Nuffel A Van Weyenberg S Maes D. Prevalence of lameness and claw lesions during different stages in thereproductive cycle of sows and the impact on reproduction results. Animal. (2013) 7:1174–81. 10.1017/S1751731113000232 23714359 PMC3666190

[5] Enokida M Sasaki Y Hoshino Y Saito H Koketsu Y. Claw lesions in lactating sows on commercial farms were associated with postural behavior but not with suboptimal reproductive performance or culling risk. Livest Sci. (2011) 136:256–61. 10.1016/j.livsci.2010.09.017

[6] Pluym L Van Nuffel A Dewulf J Cools A Vangroenweghe F Van Hoorebeke S. Prevalence and risk factors of claw lesions and lameness in pregnant sows in two types of group housing. Vet Med. (2011) 56:101–9. 10.17221/3159-VETMED

[7] Calderón Díaz JA Fahey AG Boyle LA. Effects of gestation housing system and floor type during lactation on locomotory ability; body, limb, and claw lesions; and lying-down behavior of lactating sows1. J Anim Sci. (2014) 92:1675–85. 10.2527/jas.2013-6279 24663161

[8] Nalon E Conte S Maes D Tuyttens FAM Devillers N. Assessment of lameness and claw lesions in sows. Livest Sci. (2013) 156:10–23. 10.1016/j.livsci.2013.06.003

[9] Amstel Sv. Claw horn growth and wear rates, toe length, and claw size in commercial pigs: a pilot study. J. Swine Health Prod. (2010) 18:239–43.

[10] Kramer T Donin DG Tomasi PHD Fireman A Fernandes SR Teixeira A. Prevalence and severity of claw lesions in sows in intensive systems in Brazil. Semina Ciênc Agrár. (2023) 44:301–16. 10.5433/1679-0359.2023v44n1p301

[11] Shearer JK. Hoof wall cracks in cattle. EDIS. (2007) 19:1–2. 10.32473/edis-vm131-2007

[12] Hultén F Lundeheim N Dalin A-M Einarsson S. A field study on group housing of lactating sows with special reference to sow health at weaning. Acta Vet Scand. (1995) 36:201–12. 10.1186/BF03547689 7484547 PMC8095448

[13] Jørgensen B Andersen S. Genetic parameters for osteochondrosis in Danish Landrace and Yorkshire boars and correlations with leg weakness and production traits. Anim Sci. (2000) 71:427–34. 10.1017/S1357729800055442

[14] Lisgara M. Hoof lesions and lameness in sows in three Greek swine herds. J Swine Health Prod. (2015) 23:244–51.28405432

[15] Associação brasileira de indústria produtora e exportação de carne suína. Exportação Mundial de Carne suí*na* (2011). Available online at: http://www.abipecs.org.br/pt/estatisticas/mundial/exportacao.html (accessed June 18, 2024).

[16] Anil SS Anil L Deen J Baidoo SK Walker RD. Factors associated with claw lesions in gestating sows. J Swine Health Prod. (2007) 15:78–83.

[17] van Riet MMJ Vangeyte J Janssens GPJ Ampe B Nalon E Bos E-J. On-farm claw scoring in sows using a novel mobile device. Sensors. (2019) 19:1473. 10.3390/s19061473 30917567 PMC6470472

[18] Volkmann N Kulig B Hoppe S Stracke J Hensel O Kemper N. On-farm detection of claw lesions in dairy cows based on acoustic analyses and machine learning. J Dairy Sci. (2021) 104:5921–31. 10.3168/jds.2020-19206 33663849

[19] Cañeque V Pérez C Velasco S Diaz MT Lauzurica S Álvarez I. Carcass and meat quality of light lambs using principal component analysis. Meat Sci. (2004) 67:595–605. 10.1016/j.meatsci.2004.01.002 22061809

[20] Fernandes Júnior GA Garcia DA Hortolani B de Albuquerque LG. Phenotypic relationship of female sexual precocity with production and reproduction traits in beef cattle using multivariate statistical techniques. Ital J Anim Sci. (2019) 18:182–8. 10.1080/1828051X.2018.1503570

[21] Gagaoua M Monteils V Picard B. Decision tree, a learning tool for the prediction of beef tenderness using rearing factors and carcass characteristics. J Sci Food Agric. (2019) 99:1275–83. 10.1002/jsfa.9301 30073653

[22] Destefanis G Barge MT Brugiapaglia A Tassone S. The use of principal component analysis (PCA) to characterize beef. Meat Sci. (2000) 56:255–9. 10.1016/S0309-1740(00)00050-4 22062076

[23] Murtagh F Contreras P. Algorithms for hierarchical clustering: an overview. WIREs Data Min Knowl Discov. (2012) 2:86–97. 10.1002/widm.53

[24] Young MG Tokach MD Aherne FX Main RG Dritz SS Goodband RD. Comparison of three methods of feeding sows in gestation and the subsequent effects on lactation performance1. J Anim Sci. (2004) 82:3058–70. 10.2527/2004.82103058x 15484959

[25] Sauvant D Schmidely P Daudin JJ St-Pierre NR. Meta-analyses of experimental data in animal nutrition. Animal. (2008) 2:1203–14. 10.1017/S1751731108002280 22443733

[26] Lovatto PA Lehnen CR Andretta I Carvalho AD Hauschild L. Meta analysis in scientific research: a methodological approach. Rev Bras Zootec. (2007) 36:285–94. 10.1590/S1516-35982007001000026

[27] Mevik BH Wehrens R. Introduction to the pls Package. Help Section of the “Pls” Package of R Studio Software. Ontario: Ontario Pork Industry Council (2015). p. 1–23.

[28] Liu C Zhang X Nguyen TT Liu J Wu T Lee E. Partial least squares regression and principal component analysis: similarity and differences between two popular variable reduction approaches. Gen Psychiatr. (2022) 35:e100662. 10.1136/gpsych-2021-100662 35146334 PMC8796256

[29] Kroustallas FG Papadopoulos GA Skampardonis V Leontides L Fortomaris P. Monitoring claw length, feet infrared temperature, mobility and backfat tissue changes in replacement gilts of different genetic lines in three farrow-to-finish herds in Greece. Vet Sci. (2023) 10:199. 10.3390/vetsci10030199 36977238 PMC10051576

[30] Lemmens L Schodl K Fuerst-Waltl B Schwarzenbacher H Egger-Danner C Linke K. The combined use of automated milking system and sensor data to improve detection of mild lameness in dairy cattle. Animals. (2023) 13:1180. 10.3390/ani13071180 37048436 PMC10093521

[31] Kraeling RR Webel SK. Current strategies for reproductive management of gilts and sows in North America. J Anim Sci Biotechnol. (2015) 6:1–14. 10.1186/2049-1891-6-3 25838898 PMC4382856

[32] Klein A Kramer T Cornelison AS Schweer WP Langer S Rapp C. The effects of day of mixing post-insemination, flooring type, and gilt replacement protocol on the severity of claw lesions of sows: a case study using logistic regression. Animal Sci Proc. (2023) 14:824–5. 10.1016/j.anscip.2023.09.012

[33] Henningsen DJ. Characterization of Toe and Dew Claw Overgrowth in Swine Breeding Herds. Ames, IA: Iowa State University (2023). Available online at: https://www.proquest.com/docview/2859578982/abstract/477476853134F82PQ/1 (accessed March 5, 2024).

[34] Perini JEGN Ludtke CB Carmo N Peripolli V Tanure CBGS Seixas L. Housing system during pregnancy on behavior, reproductive and health parameters of sows. Arch Zootec. (2021) 70:259–69.28405441

[35] Hartnett P Boyle LA O'Driscoll K. Rearing in female-only groups and dietary mineral supplementation improves sow welfare in the early parities and lifetime performance. Transl Anim Sci. (2020) 4:txaa176. 10.1093/tas/txaa176 33367220 PMC7745001

[36] Lütke-Dörhoff M Schulz J Westendarp H Visscher C Wilkens MR. Comparative study of the effects of two dietary sources of vitamin D on the bone metabolism, welfare and birth progress of sows fed protein- and phosphorus-reduced diets. Animals. (2022) 12:1678. 10.3390/ani12131678 35804577 PMC9265063

[37] KilBride AL Gillman CE Green LE. A cross-sectional study of prevalence and risk factors for foot lesions and abnormal posture in lactating sows on commercial farms in England. Anim Welf. (2010) 19:473–80. 10.1017/S0962728600001950

[38] Webb NG Penny RH Johnston AM. Effect of a dietary supplement of biotin on pig hoof horn strength and hardness. Vet Rec. (1984) 114:185–9. 10.1136/vr.114.8.185 6710832

[39] Gillman CE KilBride AL Ossent P Green LE. A cross-sectional study of the prevalence of foot lesions in post-weaning pigs and risks associated with floor type on commercial farms in England. Prev Vet Med. (2009) 91:146–52. 10.1016/j.prevetmed.2009.05.023 19545923

[40] Bos EJ Maes D Riet MMJ van Millet S Ampe B Janssens GPJ. Locomotion disorders and skin and claw lesions in gestating sows housed in dynamic versus static groups. PLoS ONE. (2016) 11:e0163625. 10.1371/journal.pone.0163625 27680675 PMC5040397

[41] Kirk RK Svensmark B Ellegaard LP Jensen HE. Locomotive disorders associated with sow mortality in danish pig herds. J Vet Med Ser A. (2005) 52:423–8. 10.1111/j.1439-0442.2005.00747.x 16176574

[42] Engblom L Lundeheim N Strandberg EP Schneider M Dalin AM Andersson K. Factors affecting length of productive life in Swedish commercial sows1. J Anim Sci. (2008) 86:432–41. 10.2527/jas.2007-0310 17998436

[43] Schwertz CI Bianchi RM Cecco BS Pavarini SP Driemeier D. Causes of death of sows in three Brazilian pig farms. Pesqui Veterinária Bras. (2021) 41:e06857. 10.1590/1678-5150-pvb-6857

[44] Mehmood T Iqbal M Hassan R. Prediction of antibacterial activity in ionic liquids through FTIR spectroscopy with selection of wavenumber by PLS. Chemom Intell Lab Syst. (2020) 206:104124. 10.1016/j.chemolab.2020.104124

[45] Andersen CM Bro R. Variable selection in regression-a tutorial. J Chemom. (2010) 24:728–37. 10.1002/cem.1360

[46] van Riet MMJ Millet S Aluwé M Janssens GPJ. Impact of nutrition on lameness and claw health in sows. Livest Sci. (2013) 156:24–35. 10.1016/j.livsci.2013.06.00529767794

